# Climate signal age effects in *Pinus uncinata* tree-ring density data from the Spanish Pyrenees

**DOI:** 10.1007/s00468-024-02598-3

**Published:** 2025-01-15

**Authors:** Sophie Spelsberg, Ulf Büntgen, Inga K. Homfeld, Marcel Kunz, Edurne Martinez del Castillo, Ernesto Tejedor, Max Torbenson, Emanuele Ziaco, Jan Esper

**Affiliations:** 1 Department of Geography, Johannes Gutenberg University, 55099 Mainz, Germany; 2 Oeschger Centre for Climate Change Research, University of Bern, 3012 Bern, Switzerland; 3 Global Change Research Institute of the Czech Academy of Sciences, 60300 Brno, Czech Republic; 4 Department of Geography, University of Cambridge, Cambridge, CB2 3EN UK; 5 Faculty of Science, Department of Geography, Masaryk University, 61137 Brno, Czech Republic; 6 Department of Geology, National Museum of Natural Sciences-Spanish National Research Council (MNCN-CSIC), Madrid, Spain

**Keywords:** Climate change, Paleoclimate, Climate reconstruction, Tree line, Dendrochronology

## Abstract

**Key message:**

**The temperature sensitivity of maximum latewood density measurements in pine trees from a high-elevation site in the Spanish Pyrenees increases with tree age. Detrending modulates the intensity of the effect. **

**Abstract:**

Tree-rings are the prime archive for high-resolution climate information over the past two millennia. However, the accuracy of annually resolved reconstructions from tree-rings can be constrained by what is known as climate signal age effects (CSAE), encompassing changes in the sensitivity of tree growth to climate over their lifespans. Here, we evaluate CSAE in *Pinus uncinata* from an upper tree line site in the Spanish central Pyrenees, Lake Gerber, which became a key location for reconstructing western Mediterranean summer temperatures at annual resolution. We use tree-ring width (TRW) and maximum latewood density (MXD) measurements from 50 pine trees with individual ages ranging from 7 to 406 years. For MXD, temperature sensitivity increases significantly (*p* < 0.01) with tree age from *r* = 0.31 in juvenile rings with a cambial age < 100 years to *r* = 0.49 in adult rings > 100 years. Similar CSAE are not detected in TRW, likely affected by the overall lower temperature signal (*r*_TRW_ = 0.45 *vs. r*_MXD_ = 0.81 from 1951 to 2020). The severity of CSAE is influenced by the approach used to remove ontogenetic trends, highlighting the need to assess and consider potential biases during tree-ring standardization. Our findings reveal CSAE to add uncertainty in MXD-based climate reconstructions in the Mediterranean. We recommend studying CSAE by sampling diverse age classes in dendroclimatic field campaigns.

**Supplementary Information:**

The online version contains supplementary material available at 10.1007/s00468-024-02598-3.

## Introduction

In times of a changing climate, reliable information on past climatic conditions is invaluable (Esper et al. 2024). However, systematic meteorological measurements are temporally limited, not covering more than a century in most parts of the world (Rennie et al. 2014). Only paleoclimate records allow us to look further back in time. For the past two millennia, this paleo climate data usually stem from tree-rings. Due to their ability to record annually resolved climatic information, and because they are found almost everywhere in the world (St. George 2014), tree-rings are the most widely used climate archive for climate reconstructions back to Roman times (PAGES 2k Consortium 2013).

Dendroclimatic reconstructions assume that trees record climate in a stable manner throughout their lifespan, but this assumption has been challenged (Esper et al. 2008). Several studies have found differing climatic responses between young and old trees (Carrer and Urbinati 2004; Dorado Liñán et al. 2012; Gallardo et al. 2022; Konter et al. 2016), a phenomenon coined as climate signal age effects (CSAE; Esper et al. 2008). CSAE occur when trees show an age-related change in their response to climate—either as a stronger (or weaker) sensitivity to a certain climatic parameter, or as a shifted seasonality of the climate signal.

CSAE have been related to age-dependent changes in tree physiology which, in turn, influence the interactions between the plant and the environment (Voelker 2011). As trees go through different life stages, they are exposed to different climatic and environmental stressors (Ruiz-Benito et al. 2015), experience varying levels of competition for resources (Marques et al. 2021), and undergo substantial changes in their xylem hydraulic structure (Prendin et al. 2018), all factors that can affect tree sensitivity to climate (Carrer and Urbinati 2004, Li et al. 2013; Madrigal-Gonzales and Zavala 2014; Babushkina et al. 2015). Such a change in climate response entails consequences for long-term reconstructions. For example, tree-rings covering the calibration period with instrumental data over the most recent decades typically have a higher cambial age than those in the early reconstruction period. If the temporal distribution of ring age is not considered (see Esper et al. 2016 and Ljungqvist et al. 2020 for an overview), a reconstruction may significantly over- or underestimate the reconstructed values in some periods. It was therefore recommended to assess tree-ring based climate reconstructions for possible CSAE to evaluate and potentially mitigate systematic biases (Esper et al. 2008).

Despite the potentially negative effects on tree-ring based reconstructions, information on CSAE is still scarce. Studies on maximum density (MXD) data are missing in particular, as most recent papers focused on tree-ring width (TRW) (Copenheaver et al. 2011; Esper et al. 2008; Gallardo et al. 2022; Yu et al. 2008). In comparison to TRW, MXD generally contains stronger climatic signals, which makes it a key proxy for millennial-length climate reconstructions (Esper et al. 2012). Detailed studies using MXD exist only for the boreal region (Konter et al. 2016), whereas in the Mediterranean CSAE in MXD has been assessed only in a single study excluding young life stages and comparing two distant age groups (Dorado Liñán et al. 2012). Therefore, only limited conclusions can be drawn about CSAE in MXD in the Mediterranean region.

We here compare CSAE in MXD and TRW data from a highly replicated tree line site including various age classes in the Spanish Pyrenees. We first analyze the climate response of the two proxies and then assess the signals of individual trees as a function of their age. MXD and TRW chronologies were recently developed as part of the hemispheric MONOSTAR project (http://www.monostar.org) and update the long Lake Gerber records towards present. Gerber is one of the most important sites for temperature reconstructions in southern Europe providing annually resolved temperature estimates back to the twelfth century (Büntgen et al. 2010, 2017, 2024). Gerber is thus a key site for studying climate change in a region that is most threatened by rising temperatures (Gao and Giorgi 2008; Giorgi 2006).

## Materials and methods

### Sampling site and tree-ring data

**Fig. 1:**
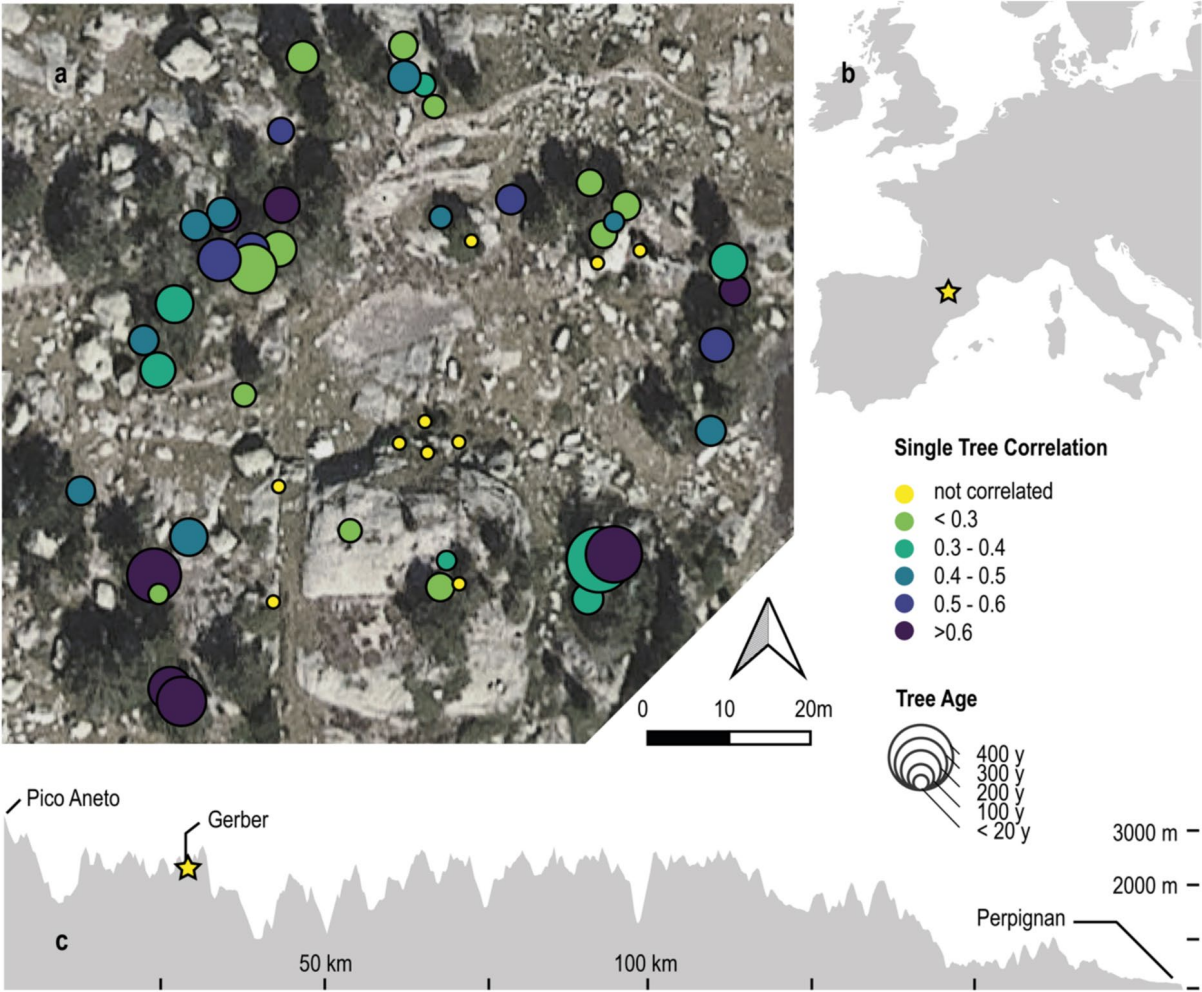
Tree line site at lake Gerber in the Spanish Pyrenees. **a** Orthophoto including 50 sampled trees (circles). Colors indicate correlations between MXD series and MJ&AS (May, June, August and September) mean temperatures from 1951 to 2020. Circle size indicates tree age. **b** Geographical location of Gerber (yellow star), and c height profile of the Pyrenees from Pico Aneto to Perpignan at sea level

**Fig. 2:**
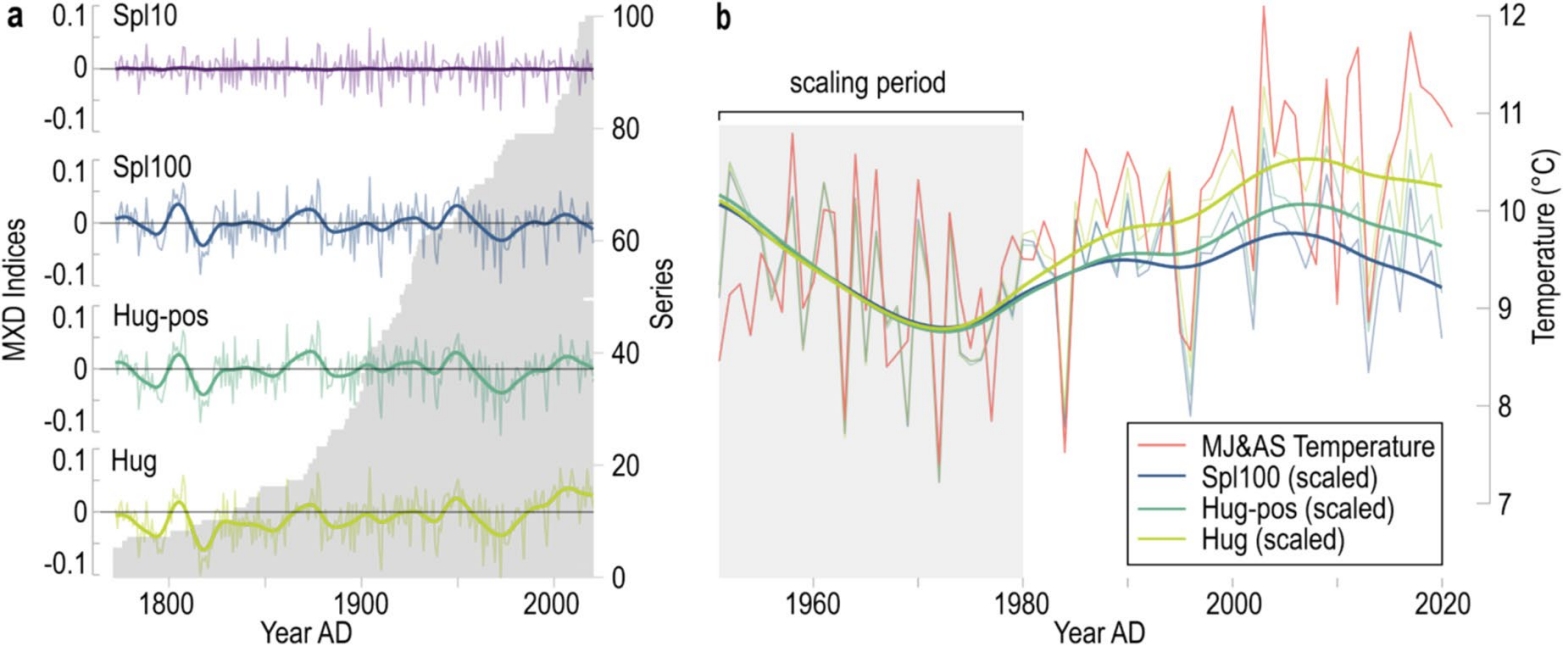
Differently detrended MXD chronologies. **a** Four chronologies derived from Hugershoff detrending without positive slopes (Hug), Hugershoff detrending with positive slopes (Hug-pos), 100-year cubic smoothing spline detrending (Spl100), and 10-year spline detrending (Spl10). Bold curves are 20-year splines. Grey bars in the background illustrate the temporal coverage of the individual measurement series. **b** The Hug, Hug-pos and Spl100 chronologies after scaling from 1951 to 1980 to MJ&AS mean temperatures (shown in red)

The Lake Gerber site is located in the Parc National d’Aigüestortes i Estany de Sant Maurici in the Central Pyrenees on a plateau at 2300 m a.s.l., ~ 100 m below the local tree line (Fig. 1). The area is covered by a sparse forest including mountain pine (*Pinus uncinata* Ramond ex DC.) as the dominant tree line species (Büntgen et al. 2010). Mean annual temperature at this high elevation is 5°C, and the temperature amplitude ranges from 1.47°C in February to 13.3°C in July. The climate is humid throughout the whole year (1185 mm annual precipitation) including minor drops during winter and high summer. Both annual and warm season mean temperatures gradually increased since the beginning of the calibration period (annual mean 1951–1980 = 4.3°C, 1991–2020 = 5.6 °C) and sharply rose since the 1980s (Fig. 2).

Two increment cores were taken at breast height from 50 living trees in a pre-defined plot. For each sampled tree, diameter at breast height (DBH) and tree height were recorded. Samples were prepared using a microtome and TRW measured on a LinTab measurement table with a precision of 0.01 mm. The measurement series were visually cross-dated and the dating verified using the COFECHA software (Holmes 1983). MXD of the same cores was measured using a WALESCH 2003 X-ray densitometer (Eschbach et al. 1995). Pith offsets were estimated considering the curvature of innermost rings on core samples (Esper et al. 2003). The final dataset extends well over the past two centuries including trees ranging 7 to 406 years (see the grey bars in Fig. 2a).

To assess the influence of different detrending techniques on CSAE, we developed five chronologies for each proxy. For MXD, this included Hugershoff fits without positive slopes (Hug), Hugershoff fits with positive slopes (Hug-pos), 100-year cubic smoothing splines (Spl100), and 10-year cubic smoothing spline (Spl10, Fig. 2). For TRW, negative exponential curves without positive slopes (Negex), negative exponential curves with positive slopes (Negex-pos), Spl100 and Spl10 were applied (Cook and Peters 1981). Additionally, results were compared to a chronology detrended with an age-dependent cubic smoothing spline adjusted to 0.67 of the individual series’ length (Spl-agedep). All indices were calculated as residuals after power transformation to avoid potentially inflated values toward the series’ end (Cook and Peters 1997) and chronologies produced using the arithmetic mean. The inter-series correlation (Rbar) and expressed population signal (EPS) were calculated to estimate covariance and the period over which the chronologies represent regional signals (Wigley et al. 1984). Detrending and chronology development were conducted using the Dendrochronology Program Library in R (dplR) (Bunn 2008) and the ARSTAN program (Cook 1985; Cook et al. 2017).

### Instrumental data and climate signal assessment

Climate correlations were computed from 1951 to 2020 using gridded precipitation and temperature from the SPREAD and STEAD compilations (Serrano Notivoli et al. 2016, 2019a, b). Those two datasets provide daily gridded precipitation (SPREAD) and minimum and maximum temperature (STEAD) values for the Iberian Peninsula at a spatial resolution of 5 × 5 km. We derived monthly means from the daily data and additionally used the Palmer Drought Severity Index (PDSI) from the KNMI Climate Explorer based on CRU TS 4.06 (Van der Schrier et al. 2013; Barichivich et al. 2022).

We evaluated tree-ring climate response using the R package treeclim considering monthly climate data from previous year January to current year October (Zang and Biondi 2015). Seasonal means from April to May (AM), May to September (MJJAS) and May to June and August to September (MJ&AS) as used in Büntgen et al. (2017) were additionally considered. The temporal stability of climatic signals was assessed by comparing findings from 1951 to 1985 and 1986 to 2020. Large-scale spatial correlation patterns were analyzed using the KNMI Climate Explorer and gridded CRU TS 4.06 land temperature data (Harris et al. 2020; Trouet and Van Oldenborgh 2013).

CSAE were assessed on a tree-by-tree basis as introduced by Carrer and Urbinati (2004) and Konter et al. (2016). This method was chosen as pooled age classes, such as considered in several recent studies (Dorado Liñán et al. 2012; Esper et al. 2008; Gallardo et al. 2022; Yu et al. 2008) may hamper comparability of varying age class limits. We here calculated climate correlations for all series covering 40 or more years of the 1951–2020 calibration period. This criterion was fulfilled by 80 of the 100 TRW series and 78 of the 100 MXD series. 68 of these TRW series and 65 of these MXD series cover the entire calibration period. The final dataset used for CSAE included trees with ages ranging from 49 to 406 years considering the pith offset estimates. When ages of tree-rings are indicated, they refer to the cambial age of the rings, i.e., the tree’s age when the rings were formed.

Since tree ages were not normally distributed according to a Shapiro–Wilk test, we used Spearman correlations to assess CSAE. Regressions were fit to log-transformed correlation results and then back-transformed for illustration. The same procedure was applied to the diameter at breast height (DBH) and tree height data to investigate their impact on tree-ring climate sensitivity. We finally assessed the percentages of series correlating significantly with MJ&AS temperatures in a moving window approach. Correlations and regressions were considered statistically significant for associated *p*-values < 0.05.

## Results and discussion

### Covariance and chronology climate signals

The raw data reach mean inter-series correlations (Rbar) of 0.29 in MXD and 0.21 in TRW. After removal of ontogenetic trends, these values rise to 0.30 and 0.25 in the detrended MXD and TRW chronologies, respectively; the expressed population signal (EPS) remains > 0.85 in all chronologies back to 1850. The various detrendings substantially alter serial correlation so that first-order autocorrelations (AC1) range from  – 0.40 to 0.29 in MXD and  – 0.33 to 0.35 in TRW.

**Fig. 3:**
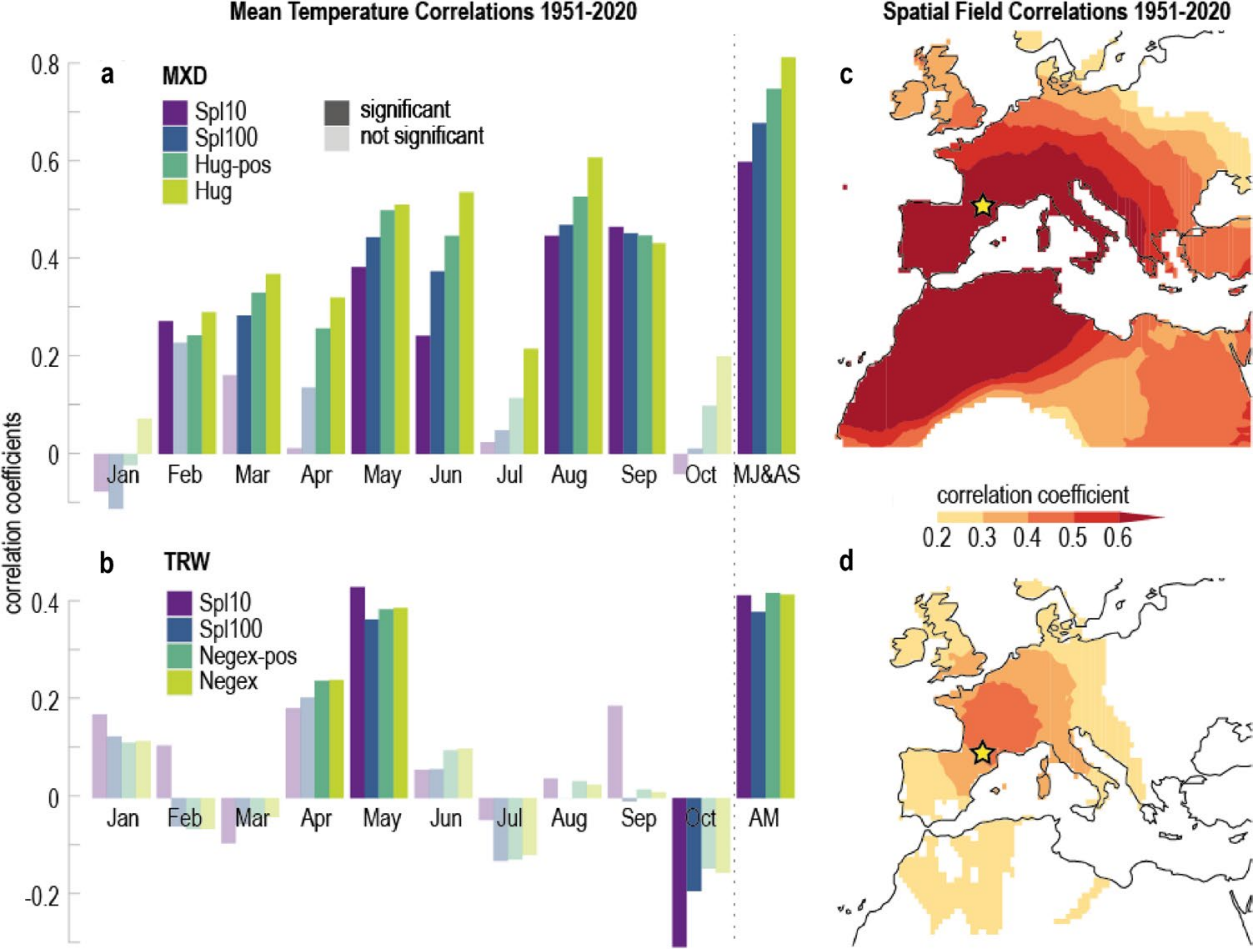
MXD and TRW climate responses. Correlations of **a** four MXD chronologies and **b** four TRW chronologies from 1951 to 2020 with monthly temperatures from January to October, and MJ&AS and AM seasons. **c** Spatial correlation patterns of the MXD Hug chronology against MJJAS temperatures, and d the TRW Negex chronology against AM temperatures from 1951 to 2020

As discussed in Büntgen and Esper (2024), the correlations between MXD and gridded temperature data reveal a bi-modal pattern from May–June and August–September (MJ&AS). Depending on the detrending, the correlation coefficient reaches *r* = 0.81, which is an exceptionally strong climatic signal even at hemispheric scale (Esper et al. 2016). Monthly resolved climate–growth correlations are also strongest for those four months, with a gap in July which does not exceed *p* > 0.05 (Fig. 3). The bi-modal signal is related to exceptionally warm conditions in July (Royo-Navascues et al. 2022) and has been reported in previous studies from the Mediterranean (Büntgen et al. 2017; Camarero et al. 2010; Esper et al. 2020). Our comparison over independent calibration and verification periods from 1951 to 1985 and 1986–2020 indicates that the MJ&AS signal is stable throughout time (Fig. S1).

For TRW, the main climatic driver is spring season temperature from April–May (AM), with the strongest correlation of *r* = 0.42 observed in the Negex-pos chronology (Fig. 3). This spring signal, opposed to the warm-season signal found in MXD, represents the onset of cambial growth occurring in April/May at this high-elevation site (Camarero et al. 1998). However, the AM temperature signal is neither stable for all detrendings nor through time (Fig. S1). At monthly resolution, only May temperature correlates significantly with all TRW chronologies. The weaker TRW temperature response is part of a mixed signal associated with regional drought (Royo-Navascues et al. 2022) constraining the skill of climate reconstructions from *Pinus uncinata* TRW data in the Pyrenees (Seim et al. 2015). Our findings thus confirm a weaker association between climate and TRW chronologies, compared to MXD chronologies, making the latter the most important proxy for temperature reconstructions in the Mediterranean (Büntgen et al. 2010).

### Climate signal age effects

**Fig. 4:**
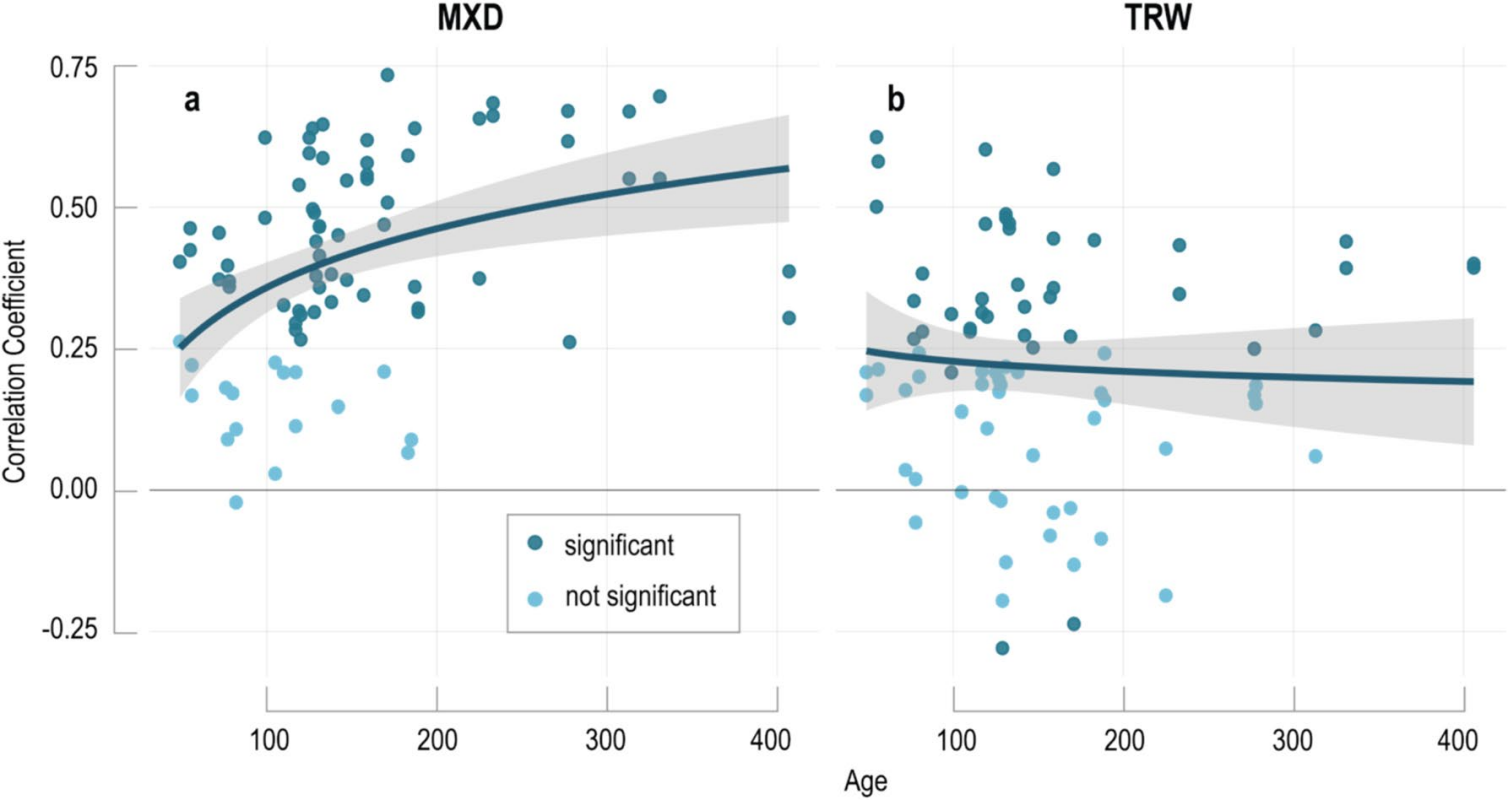
CSAE in MXD and TRW. **a** Single-tree MXD correlations with MJ&AS mean temperatures. Each dot represents a single MXD series aligned by the cambial age of the outermost ring. Insignificant correlations are shown in light blue. Blue curve is a logarithmic regression with uncertainties (grey). **b** Same as in **a** but for TRW against AM temperatures

We find evidence for CSAE in Mediterranean MXD records including older rings correlating significantly stronger with MJ&AS temperatures than young ones. The sensitivity of individual tree-ring series increases with their cambial age, in the Hug-pos detrended MXD data from *r* = 0.31 in juvenile rings (cambial age < 100 years) to *r* = 0.49 in adult rings (cambial age > 100 years; Fig. 4). This increased sensitivity of older tree-ring sequences is consistent across all five detrendings. Spearman correlations indicate a monotonic relationship between cambial age and climate response, significant in the Hug-pos, Spl100, Spl10 and Spl-agedep detrended data (Hug *p*-value = 0.06). The same applies to the regression models fit to the age-aligned correlations (the bold curves in Fig. 4), i.e., all MXD detrendings show the same positive trend, yet Hug and Spl10 fall below the 95% significance level (Table 1).

**Table 1:** Chronology climate responses and statistical measures for CSAE (significant values marked with*)

Detrending	Climate response *r* 1951–2020	CSAE Rho Spearman	*p*-value Spearman	CSAE R^2^ regression	*p*-value regression
MXD Spl10	0.60*	0.28*	0.01*	0.04	0.06
MXD Spl100	0.68*	0.30*	< 0.01 *	0.08*	0.01*
MXD Hug-pos	0.75*	0.42*	< 0.01*	0.16*	< 0.01*
MXD Hug	0.81*	0.21	0.06	0.04	0.09
MXD Spl-agedep	0.69*	0.36*	< 0.01*	0.12*	< 0.01*
TRW Spl10	0.42*	– 0.03	0.76	< 0.01	0.40
TRW Spl100	0.38*	0.02	0.83	< 0.01	0.73
TRW Negex-pos	0.42*	0.11	0.32	0.01	0.31
TRW Negex	0.42*	– 0.05	0.66	< 0.01	0.59
TRW Spl-agedep	0.37*	0.09	0.43	0.01	0.36

In contrast to MXD, no age effect is observed in the TRW data (Fig. 4b). The TRW chronology and the single series show a generally lower temperature sensitivity. CSAE are absent in all TRW detrending techniques except for Negex-pos, which reveal a weak, although not statistically significant positive trend towards higher sensitivity in older trees.

The CSAE difference between MXD and TRW data at Lake Gerber is likely related to the varying climate responses of the two proxies. An overall strong climatic forcing as well as a sufficient sample replication appear fundamental to develop and detect CSAE (Esper et al. 2008; Trouillier et al. 2019). Since the correlations of TRW chronologies with temperature range from r = 0.3 to 0.4, much below the MXD correlation results, CSAE may not have been established and cannot be observed in these data.

**Fig. 5:**
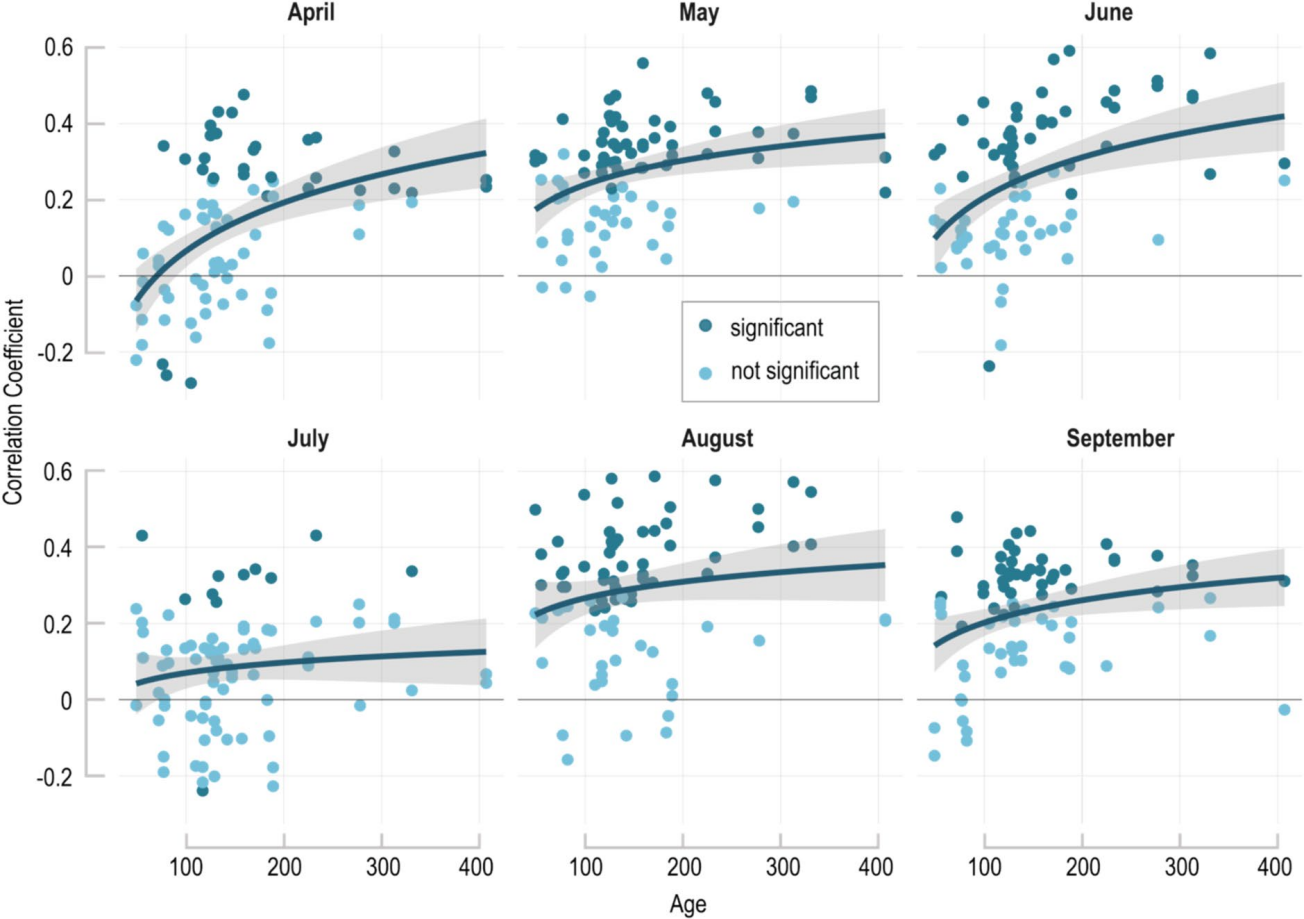
Monthly resolved CSAE in MXD. Each dot represents the correlation of a single MXD series against monthly mean temperatures from April to September. Blue curve is a logarithmic regression with uncertainties (grey)

Our results do not support a shift in the timing of the climate–growth relationship between tree-rings of young and old cambial age. Prior research revealed a delayed onset of growing seasons in old trees at alpine tree lines (Rossi et al. 2008) and temporally altered signal strength in mature tree-rings (Dorado Liñán et al. 2012). The TRW data analyzed here do not show such effects, and the MXD data indicate an opposite trend: all months with a significant climatic response show consistently increased signals in older trees (Fig. 5). This pattern is similar to that observed in CSAE studies from the Italian Alps (Carrer and Urbinati 2004).

CSAE can be explained by a combination of age-related changes in the xylem anatomical structure, tree’s physiology, and exposure to physical–mechanical stressors. As trees get older, the xylem cellular structure and its hydraulic architecture change following well defined ontogenetic trends. Young trees generally show smaller tracheids with thinner cell walls compared to old trees, which emphasizes the tree’s initial need for rapid growth and less need for structural strength (Piermattei et al. 2020). Old trees, on the other hand, show reduced photosynthetic rates and increased biomass accumulation (Stephenson et al. 2014). Most of such biomass production derive from carbon allocated to stem wood cellular walls rather than in canopy and leaves (Domec et al. 2012), and particularly in the latewood cells, which often represent the most climate-sensitive sector of tree rings (Cuny and Rathgeber 2016).

Furthermore, older and taller trees experience higher hydraulic constraints compared to small and young individuals (Carrer and Urbinati 2004). The longer xylem pathways and the need to withstand higher negative pressures (Olson et al. 2018) lead to changes in the xylem structure to prevent the risk of cavitation while preserving hydraulic efficiency (Prendin et al. 2018; Ziaco et al. 2023). Longer hydraulic pathways limit the supply of water to the canopy, forcing tall trees to close their stomata earlier and limit photosynthesis (Bond 2000; Ryan et al. 2006; Ryan and Yoder 1997), creating more stressful conditions for the trees which lead to increased climatic sensitivity (Gazol et al. 2020). Finally, young trees are more affected by wind and snow stress, hence more prone to form reaction wood (Esper et al. 2008). The physical properties of these rings differ, both in terms of size and density. All these physiological and mechanical biases may superpose climatic signals in both TRW and MXD chronologies (Gryc and Horáček 2007). However, the key role of wood anatomical structure in determining intra-annual density variability within the ring, makes these CSAE more evident in MXD data.

To further disentangle the potential effect of size-induced changes in tree physiology on climatic signal (Bond 2000; Trouillier et al. 2019), we compared climate sensitivity in MXD records against tree height, but found no significant trends (Fig. S3). This is likely due to the fact that the vertical variability of wood anatomical parameters, such as lumen area or cell wall thickness (i.e., along the stem, from the base to the treetop) is generally small for most of the stem length, while it peaks only in the first 1–2 m from the stem apex (Ziaco et al. 2023).

### Importance of detrending methodology

**Fig. 6:**
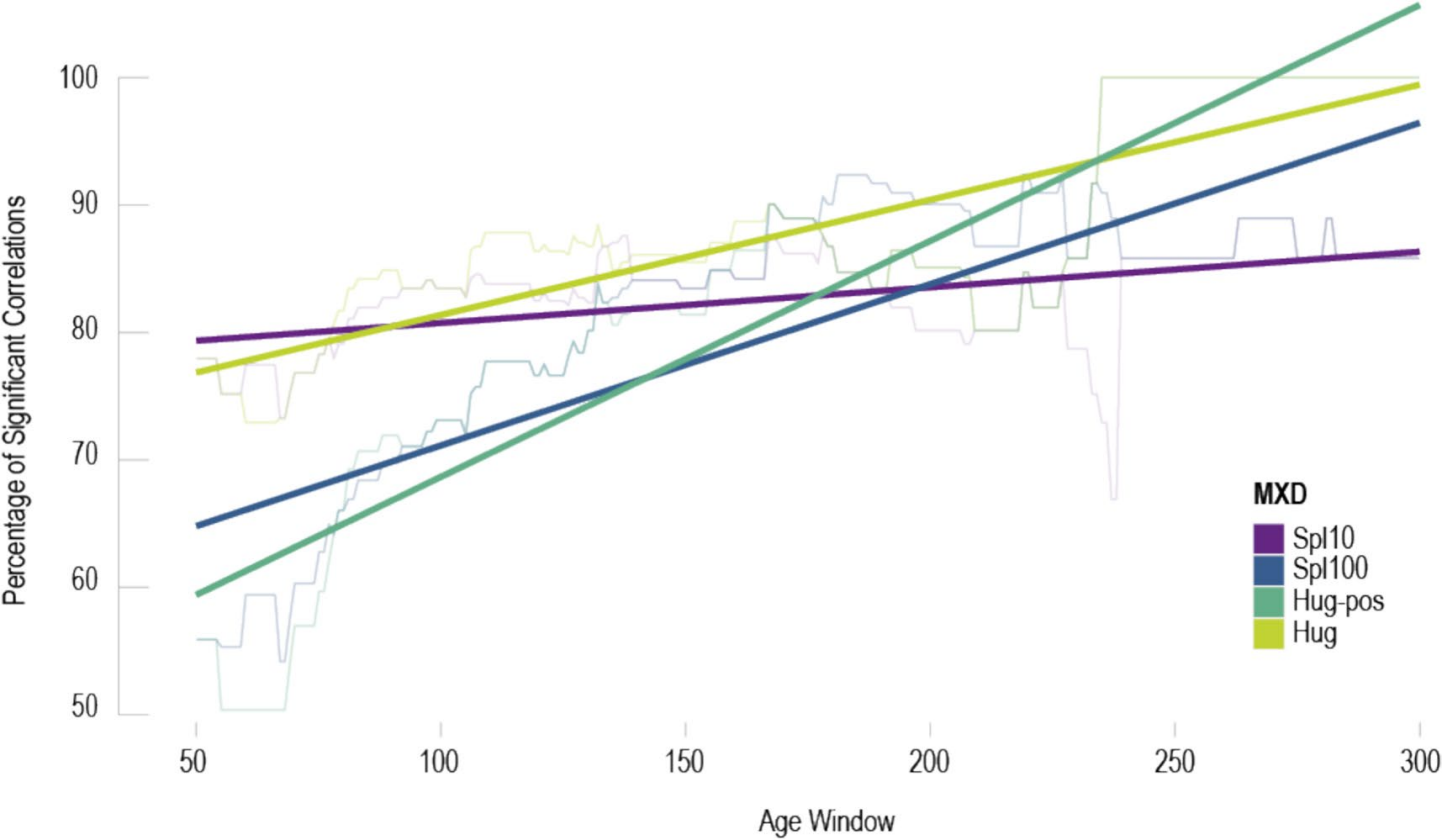
Effects of detrending on MXD CSAE. Thin curves show percentages of MXD series, aligned by tree age, correlating significantly with MJ&AS temperature from 1951 to 2020. Bold curves are linear regressions

Our findings indicate that the detrending technique may modulate CSAE. For MXD, CSAE are strongest in the data detrended using Hug-pos (Table 1; Fig. 6). In TRW, only the Negex-pos detrended data show a similar positive, though statistically not significant, trend as the MXD data (Fig. S2). These findings suggest that certain techniques enhance CSAE as is the case in MXD, or even produce (weak) CSAE as is the case in TRW. Particularly the detrending approaches that allow positive slopes, including Hugershoff detrending for MXD, appear prone to CSAE intensification.

Enhanced CSAE might result from particular detrending implementations that remove more climate signal from short than from long series. When no Hugershoff or negative exponential curve can be applied, dplR fits a linear function with a positive slope instead. As many of the raw series show a recent increase in MXD and TRW, these fits occurred in more than 50% of all cases. However, a straight line with a positive slope attenuates or even removes the recent warming signal particularly from the short series, e.g., shorter than 50 years length, whereas these effects are mitigated in the longer series. This procedure systematically lowers the climate correlations in younger trees and thereby artificially increases CSAE. The enhanced CSAE in the data detrended with an age-dependent spline can be explained similarly, as such a spline adapts much more flexibly to short series and removes more climate signal from them. Accordingly, the minor increase in climate sensitivity with age, as recorded in the TRW Negex-pos data, should be considered with caution.

In MXD, CSAE appear only slightly modified in the Hug-pos detrended data (Fig. S2). Considering the changing percentages of significant correlations, we find increasing values across all detrendings (Fig. 6). Whereas only 56–78% of trees ranging from 50 to 100 years reveal significant warm season temperature signals, these values increase to 86–100% for trees ranging from 250 to 350 years.

## Conclusions

We provide evidence for CSAE in *Pinus uncinata* maximum latewood density data from the Spanish Pyrenees. MXD data from old rings show a stronger warm season temperature signal compared to young rings. In TRW, no such trends have been recorded, partly due to the generally weaker and temporally unstable temperature signal. Further studies on CSAE in MXD data from other species are needed to make more general statements on CSAE in density data.

Although the observed effects are statistically significant in MXD, the question arises whether CSAE can artificially be induced or amplified by the detrending method. This conclusion is partly supported as the detrendings allowing fits with positive slopes showed stronger CSAE. These methods appear to remove climate (warming) signals particularly from shorter series and thereby increase the differences between young and old trees. It is therefore recommended to test different detrending methods and evaluate potentially changing CSAE before conducting climate reconstructions. Such tests require sampling strategies that include trees of varying age classes, however.

### Author contribution statement

SS and JE designed and coordinated the study. JE, UB, EMdC, MT, SS, IKH, EZ and MK did the field and laboratory work. ET processed and provided the climate data. SS performed the statistical analysis and wrote the first draft. All authors contributed to improve and discuss the manuscript.

## Supplementary Information

Below is the link to the electronic supplementary material.

Supplementary file1 (DOCX 524 KB)

## Data Availability

The tree-ring data and meta information are available at http://www.monostar.org and upon request to the authors.
