# Supplementary material for: Climate signal age effects in Pinus uncinata tree-ring density data from the Spanish Pyrenees
Source: Trees (Berl West). 2025 Jan 15;39(1):23. doi: 10.1007/s00468-024-02598-3 (PMC11735477; doi:10.1007/s00468-024-02598-3)
Supplement: Supplementary file 1 — Supplementary file1 (DOCX 524 KB) [file 468_2024_2598_MOESM1_ESM.docx]

**Supplementary materials**


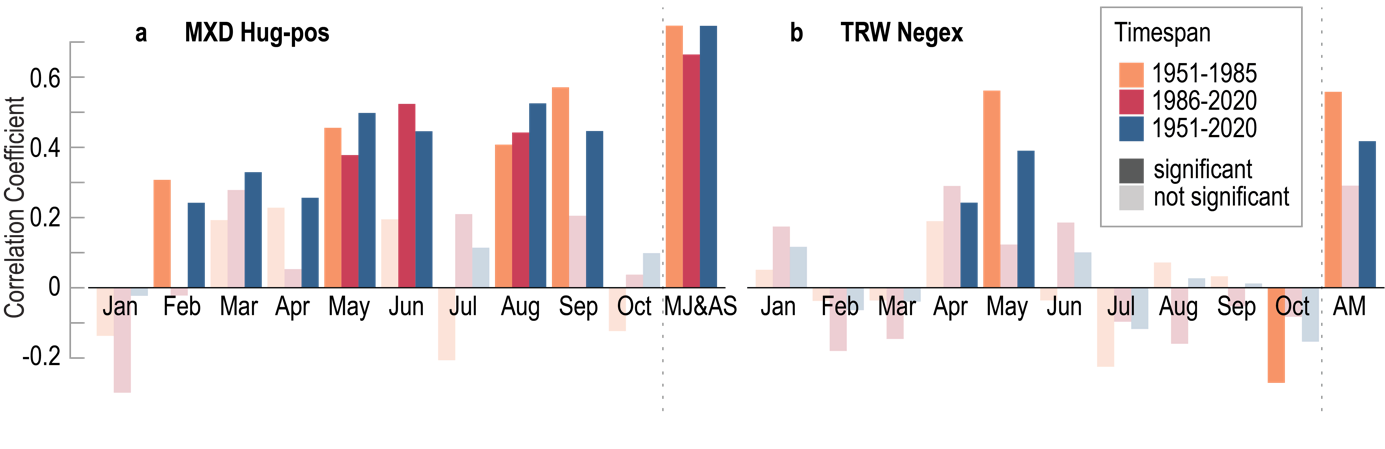


**Fig. S1**  Stability of the temperature signal. Three independently calibrated time periods were used to control the stability for (**a**) MXD and (**b**) TRW


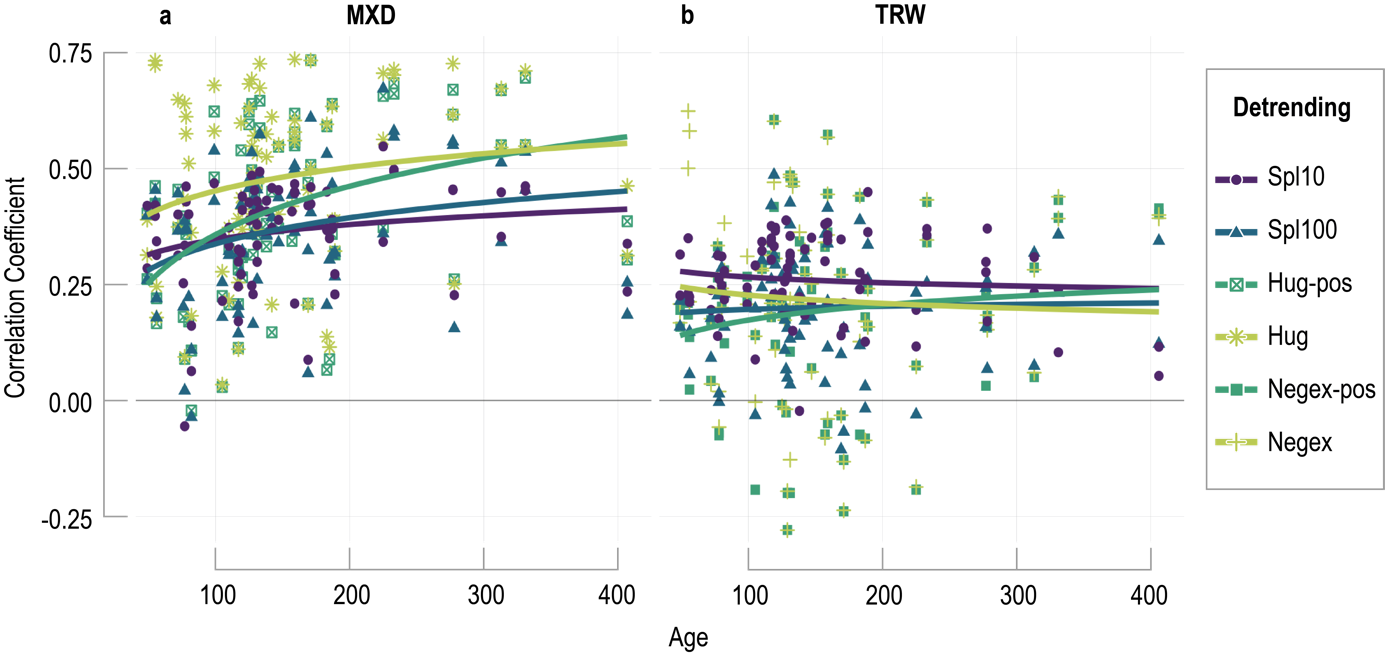


**Fig. S2** Effects of the detrending on CSAE. Each dot represents a single series, each color a detrending. Bold lines are logarithmic regressions for (**a**) MXD and (**b**) TRW


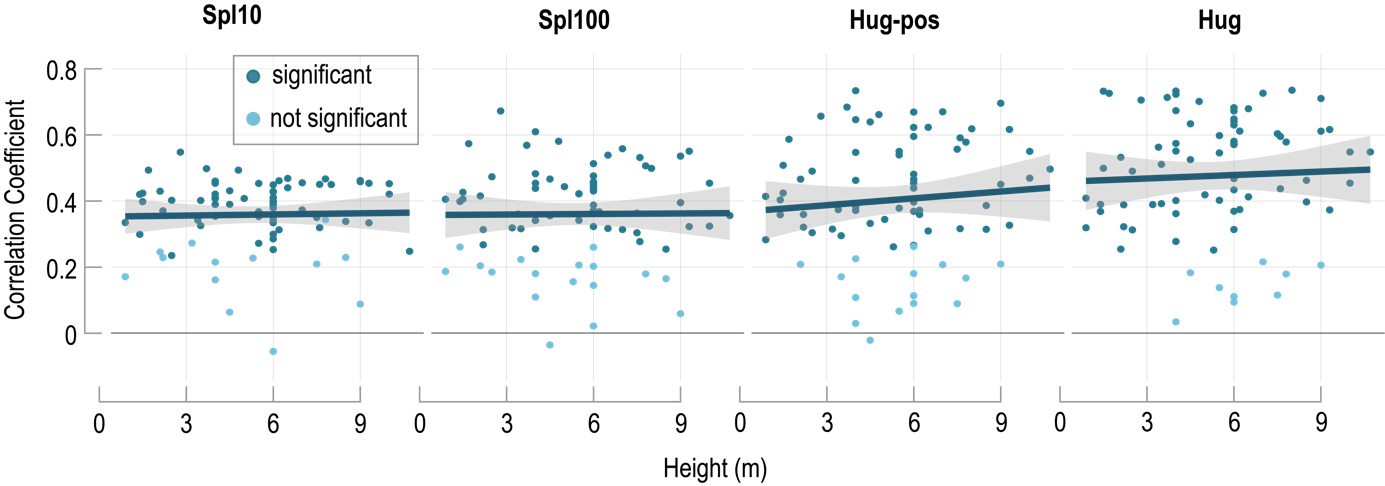


**Fig. S3** MXD MJ&AS signals plotted against tree height considering different detrending
